# Supplementary material for: What influences slum residents’ choices of healthcare providers for common illnesses? Findings of a Discrete Choice Experiment in Ibadan, Nigeria
Source: PLOS Glob Public Health. 2023 Mar 13;3(3):e0001664. doi: 10.1371/journal.pgph.0001664 (PMC10021758; doi:10.1371/journal.pgph.0001664)
Supplement: S1 Appendix — (DOCX) [file pgph.0001664.s001.docx]

**S1 Appendix**

Three Symptom Scenarios

*Please imagine the following situations*

| *Disease* | *Scenario* |
| --- | --- |
| Malaria | You woke up yesterday morning feeling feverish. You are experiencing mild headache and body ache. Before the end of the day, you started feeling cold. In the morning today, you ate and vomited and begin to feel dehydrated. There are lot of mosquitoes in your area and you have been bitten several times in the past one week. |
| Diarrhoea/Cholera | You have had diarrhoea several times in the last 24 hours. You are experiencing serious stomach cramps, which was accompanied by periodic vomiting. Your symptoms began yesterday morning and you have been drinking plenty of water yet you still feel severely dehydrated. Presently, you have started losing body weight. |
|  |  |
| Depression | You have been feeling really down for the last few weeks. You do not enjoy things the way you normally would. In fact, nothing gives you pleasure and satisfaction, and when good things happen, they do not seem to make you happy. You have to force yourself to get through the day, and the smallest tasks seem hard to do. You find it hard to concentrate on anything and have no energy at all. Though you feel tired at night, you cannot sleep, and wake up too early in the morning. You feel worthless and want to give up. You do not feel like talking and are not taking part in activities like you used to. Your family has noticed that you have not been yourself for about the last month. |
