# Supplementary material for: What influences slum residents’ choices of healthcare providers for common illnesses? Findings of a Discrete Choice Experiment in Ibadan, Nigeria
Source: PLOS Glob Public Health. 2023 Mar 13;3(3):e0001664. doi: 10.1371/journal.pgph.0001664 (PMC10021758; doi:10.1371/journal.pgph.0001664)
Supplement: S1 Table — (DOCX) [file pgph.0001664.s002.docx]

**S1 Table**

**Results of the Mixed Logit and Latent Class with 3 models for each of the scenarios**

| **Attribute** | **Level** | **Reference** | **Scenario 1** | | | **Scenario 2** | | | **Scenario 3** | | | |
| --- | --- | --- | --- | --- | --- | --- | --- | --- | --- | --- | --- | --- |
|  |  |  | **OR (mean and 95% CI)** | | | **OR (mean and 95% CI)** | | | **OR (mean and 95% CI)** | | | |
|  |  |  | **Class A** | **Class B** | **Class C** | **Class A** | **Class B** | **Class C** | **Class A** | **Class B** | **Class C** | |
| Visit an HCF? | Reference HCF | Self care | 98.9 (14.3,682) | 0.72 (0.16,3.26) | 800 (91.1,7030) |  |  |  |  |  |  | |
| Option B bias | Option B | Option A | 1.08 (0.98,1.19) | | | 0.96 (0.91,1.02) | | | 0.86 (0.79,0.93) | | | |
| Travel Time | 30-minute increase | | 0.89 (0.71,1.12) | 1.18 (0.65,2.13) | 0.39 (0.20,0.75) | 0.20 (0.08,0.52) | 1.07 (0.57,2.01) | 0.87 (0.75,1.00) | 22.4 (7.57,66.6) | 0.28 (0.00,80.8) | 148  (66.8,328.) | |
| Facility Ownership | Community | Government | 1.38 (0.89,2.14) | 1.37 (0.37,5.03) | 0.68 (0.55,0.83) | 1.14 (0.30,4.34) | 1.37 (0.85,2.21) | 0.80 (0.65,0.99) | 2.90 (1.76,4.78) | 2.15 (0.21,21.8) | 0.61 (0.52,0.71) | |
|  | Private | Government | 0.12 (0.05,0.26) | 10.2 (2.20,47.3) | 0.63 (0.45,0.89) | 0.39 (0.08,1.84) | 0.00 (0.00,0.04) | 1.26 (1.06,1.50) | 0.68 (0.46,1.01) | 0.03 (0.00,0.36) | 1.52 (1.15,2.00) | |
| Drug availability | Partial | None | 1.79 (1.42,2.24) | 1.01 (0.59,1.73) | 0.84 (0.51,1.39) | 2.26 (0.74,6.90) | 1.10 (0.74,1.63) | 1.13 (1.00,1.29) | 0.05 (0.03,0.10) | 1.01 (7.66,0.00) | 1.82 (1.44,2.30) | |
|  | Full | None | 2.16 (1.59,2.94) | 1.16 (0.65,2.04) | 1.43 (0.77,2.65) | 0.83 (0.22,3.09) | 0.66 (0.37,1.20) | 2.01 (1.60,2.51) | 1.83 (1.25,2.67) | 1.39 (0.35,5.55) | 1.25 (1.09,1.42) | |
| Privacy | Yes | No | 0.92 (0.72,1.16) | 0.97 (0.67,1.39) | 1.25 (1.07,1.45) | 1.38 (0.54,3.50) | 0.71 (0.45,1.12) | 1.15 (1.00,1.32) | 2.64 (1.95,3.56) | 2.84 (0.66,12.1) | 1.67 (1.40,2.00) | |
| Gender Choice | Yes | No | 0.89 (0.72,1.10) | 1.21 (0.71,2.06) | 1.03 (0.81,1.32) | 0.45 (0.21,0.96) | 0.99 (0.68,1.44) | 0.93 (0.85,1.03) | 1.50 (1.16,1.92) | 0.50 (0.24,1.06) | 0.92 (0.85,1.00) | |
| Facility | Inpatient | Daycare | 1.08 (0.85,1.38) | 1.09 (0.66,1.80) | 0.91 (0.78,1.07) | 2.04 (1.02,4.06) | 2.01 (1.32,3.04) | 0.90 (0.77,1.05) | 0.77 (0.62,0.94) | 3.86 (0.77,19.3) | 0.89 (0.78,1.02) | |
| Staff Qualification | Doctor | Drug Vendor | 1.16 (0.63,2.13) | 1.72 (1.03,2.87) | 1.69 (1.05,2.73) | 0.19 (0.04,0.79) | 1.33 (0.72,2.44) | 1.82 (1.59,2.09) | 0.63 (0.36,1.08) | 7.33 (8.41,6.40) | 1.00 (0.79,1.25) | |
| Cost | 1000-Naira increase | | 0.92 (0.82,1.03) | 1.04 (0.92,1.18) | 0.76 (0.65,0.89) | 0.57 (0.41,0.80) | 0.89 (0.76,1.05) | 0.96 (0.92,1.00) | 3.43 (1.71,6.88) | 0.19 (0.05,0.73) | 1.11 (0.88,1.40) | |
| Adj Rho^2 |  |  | 0.4637 | | | 0.4068 | | | 0.4495 | | | |
| AIC |  |  | 5907.26 | | | 6534.46 | | | 6063.51 | | | |
| % in class |  |  | 38.9% | 10.6% | 50.5% | 3.1% | 25.2% | 71.7% | 37.0% | 1.4% | 61.6% | |
| High Income |  |  | 0.59 (0.28,1.23) | NA | 0.70 (0.31,1.56) | 1.91 (0.67,5.45) | NA | 1.49 (0.98,2.27) | 1.41 (0.38,5.17) | NA | 1.80 (0.49,6.50) | |
| High Education |  |  | 0.53 (0.24,1.19) | NA | 1.28 (0.60,2.73) | 1.89 (0.66,5.40) | NA | 0.74 (0.47,1.16) | 0.77 (0.22,2.69) | NA | 0.96 (0.28,3.31) |  |
| Female |  |  | 1.23 (0.60,2.50) | NA | 0.36 (0.17,0.76) | 0.39 (0.14,1.07) | NA | 1.64 (1.08,2.49) | 0.61 (0.13,2.77) | NA | 0.62 (0.13,2.76) |  |
| Large Household |  |  | 1.53 (0.71,3.27) | NA | 1.31 (0.64,2.70) | 0.79 (0.26,2.42) | NA | 0.90 (0.58,1.39) | 0.57 (0.14,2.33) | NA | 0.50 (0.12,2.04) |  |

Table S1 – Results from Latent class models with 3 classes.
