# Supplementary material for: What influences slum residents’ choices of healthcare providers for common illnesses? Findings of a Discrete Choice Experiment in Ibadan, Nigeria
Source: PLOS Glob Public Health. 2023 Mar 13;3(3):e0001664. doi: 10.1371/journal.pgph.0001664 (PMC10021758; doi:10.1371/journal.pgph.0001664)
Supplement: Attachment — Submitted filename: Response to reviewers comment PLOS Global Health.docx [file pgph.0001664.s003.docx]

**Reviewer’s comment on the manuscript**

1. full reporting of the discrete choice experiment

*Response:* Done- reworked into the methodology section

2. justification of the specific scenarios used in relation to the research question

*Response:* Done- reworked into the methodology section

3. explanation of the validity checks included, and clarity on key decision points in the analysis such as number of latent classes selected.

*Response:* Done- reworked into the methodology section

4. The Introduction should also be much more specific to the study context and research question here.

*Response*: Done- Introduction section

**Also, we responded to DCE checklist as below:**

**DCE Checklist: What influences slum residents' choices of healthcare providers for common illnesses? Findings of a Discrete Choice Experiment in Ibadan, Nigeria**

1. Was a well-defined research question stated and is conjoint analysis an appropriate method for answering it?

1.1 Were a well-defined research question and a testable hypothesis articulated?

**The study aimed to quantify the influence of service provider characteristics on respondents’ choice of healthcare provider in possible cases of general symptoms associated with malaria, diarrhoea/cholera and depression**

1.2 Was the study perspective described, and was the study placed in a particular decision-making or policy context?

**Understanding patient preferences, especially in low resource settings of LMICs is vital to designing effective healthcare policy and health systems and informing predictions of how service users will respond to proposed changes**

1.3 What is the rationale for using conjoint analysis to answer the research question?

**Findings from our multi-country study on improving health in slums revealed that treatment preference was not fully researched in literature. The DCE was found to be the most appropriate method for quantifying and understanding the respondents’ choice/preference for healthcare when presented with hypothetical symptoms,**

2. Was the choice of attributes and levels supported by evidence?

2.1 Was attribute identification supported by evidence (literature reviews, focus groups, or other scientific methods)?

**Secondary analysis from the study that explores health service access and use among people who live in slum areas in four countries, in-depth interview and literature review were used to identify the attributes ad levels.**

2.2 Was attribute selection justified and consistent with theory?

2.3 Was level selection for each attribute justified by the evidence and consistent with the study perspective and hypothesis?

**Literature review, expert opinion from health practitioners and pilot testing were used to ensure appropriateness and consistency.**

3. Was the construction of tasks appropriate?

3.1 Was the number of attributes in each conjoint task justified (that is, full or partial profile)?

**Full profiles were presented for each choice task. The complexity of the task was assessed during the piloting stage, and respondents were comfortable with making choices based on all attributes.**

3.2 Was the number of profiles in each conjoint task justified?

**For each choice task, respondents were offered two unlabelled profiles with a self-care opt-out (and a follow-up forced choice if the opt-out was chosen). Our experience during the development and piloting phases suggested that it would be counterproductive to include additional profiles. Most of the respondents had no concerns with the number of profiles.**

3.3 Was (should) an opt-out or a status-quo alternative (be) included?

**We included an opt-out option of self-care for each choice task. This was considered appropriate and realistic as in practice respondents might choose to self-treat rather than seek care when experiencing the symptoms included for each scenario.**

4. Was the choice of experimental design justified and evaluated?

4.1 Was the choice of experimental design justified? Were alternative experimental designs considered?

**We developed both orthogonal and D-efficient designs, using the specialist software NGene to identify the optimal design with each approach. With the D-efficient design, it was possible to incorporate prior information on the likely ordering of level preferences for certain attributes (cost, time and drug-availability), and therefore this design was preferred**.

4.2 Were the properties of the experimental design evaluated?

Our chosen design aimed to maximise efficiency and maintain level balance while not overburdening participants. Given the heterogeneity of available health care facilities and the conceptual independence of the attributes, we did not anticipate that any specific profiles would be implausible. We confirmed this during the extensive piloting phase in which no such concerns were reported by participants.

4.3 Was the number of conjoint tasks included in the data-collection instrument appropriate?

Each respondent was requested to complete 9 choice tasks per scenario, giving 27 altogether. This is high relative to the typical burden reported in the literature, but not beyond what is considered acceptable. Few concerns were raised during piloting that this burden was excessive. However, the few respondents who noted this completed the survey in one session.

5. Were preferences elicited appropriately, given the research question?

5.1 Was there sufficient motivation and explanation of conjoint tasks?

The research assistants ensured that the participants were aware that the study is based on hypothetical scenarios of real-life diseases. They explained that the study seeks to identify the major factors that influence their health seeking behaviour. The participants were told the meaning of each of the attributes (and their levels), and why we had to choose one in each conjoint task. Before the tasks was presented to them, each of the scenario was also read out to the participants and they were asked to state the disease. They mostly responded with malaria/fever, cholera/diarrhoea and depression5.2 Was an appropriate elicitation format (that is, rating, ranking, or choice) used? Did (should) the elicitation format allow for indifference?

We used a choice format as we felt this reflected the real world decision we were attempting to understand i.e. which (if any) health care facilities would the respondent choose when experiencing a specified symptom profile.

5.3 In addition to preference elicitation, did the conjoint tasks include other qualifying questions (for example, strength of preference,

confidence in response, and other methods)?

We chose not to include such qualifying questions as we were concerned about the cognitive burden this might impose and the challenge involved in contextualising such questions so they could be properly interpreted by respondents. We did include an opt-out question, but respondents who chose this option were asked a forced-choice question as a follow-up, in case that choice was motivated by a reluctance to choose between the hypothetical profiles.

6. Was the data collection instrument designed appropriately?

6.1 Was appropriate respondent information collected (such as sociodemographic, attitudinal, health history or status, and treatment

experience)?

We collected a range of sociodemographic and economic variables in line with our previous research experience within our target population – income, education, age, marital status, and household size.

6.2 Were the attributes and levels defined, and was any contextual information provided?

The attributes and levels were presented to the respondents and were informed that the survey involved combination of various levels and attributed in response to specific scenarios. The scenarios were read aloud and the respondents were able to identify the common illness. The respondents were then presented with the relevant choice tasks and asked what they would do if they had the common illness.

6.3 Was the level of burden of the data-collection instrument appropriate? Were respondents encouraged and motivated?

7. Was the data-collection plan appropriate?

7.1 Was the sampling strategy justified (for example, sample size, stratification, and recruitment)?

We provide a justification for our recruitment and sample size in the manuscript

7.2 Was the mode of administration justified and appropriate (for example, face-to-face, pen-and-paper, web-based)?

The survey was uploaded on Open Data Kit (ODK) data collection tool and administered face-to-face by a research assistant. Our previous study and reports from the pilot testing show that the mode of administration was engaging and participatory.

7.3 Were ethical considerations addressed (for example, recruitment, information and/or consent, compensation)?

All respondents who participated in the study (qualitative and quantitative) were those who had consented to being recontacted in the previous study. Nevertheless, informed consent was sought again after the study had been explained to them.

8. Were statistical analyses and model estimations appropriate?

8.1 Were respondent characteristics examined and tested?

The respondent characteristics such as income, education, age, marital status, and household size were examined and tested. They were also a fair representation of the target population.

8.2 Was the quality of the responses examined (for example, rationality, validity, reliability)?

We included a validity question within each block where we would expect a certain choice a priori. We also tested our results for option ordering bias. The results from these checks are included in the manuscript.

8.3 Was model estimation conducted appropriately? Were issues of clustering and subgroups handled appropriately?

In order to explore patient heterogeneity, we fitted both mixed-logit and latent class models. Model selection was based on the Akaike Information Criterion. This included the number of classes included in the latent class model. We fitted both 2-class and 3-class models, and the AIC suggested the improved fit from the 3-class model was marginal and not strong enough to justify the extra model complexity involved.

9. Were the results and conclusions valid?

9.1 Did study results reflect testable hypotheses and account for statistical uncertainty?

Our results directly respond to our research question by demonstrating clearly that respondents look beyond cost and convenience when choosing a health care facility. Our analysis further strongly supports the presence of preference heterogeneity for key attributes, and provides some evidence on the characteristics associated with differing preferences.

9.2 Were study conclusions supported by the evidence and compared with existing findings in the literature?

Each of the study findings/conclusions was compared with most recent existing findings in other literature.

9.3 Were study limitations and generalizability adequately discussed?

Yes. This was presented in the last paragraph in the discussions section.

10. Was the study presentation clear, concise, and complete?

10.1 Was study importance and research context adequately motivated?

Yes.

10.2 Were the study data-collection instrument and methods described?

Yes. These was discussed in details in the methods section.

10.3 Were the study implications clearly stated and understandable to a wide audience?

Yes. The conclusion summarizes this articulately.
