# Supplementary material for: What influences slum residents’ choices of healthcare providers for common illnesses? Findings of a Discrete Choice Experiment in Ibadan, Nigeria
Source: PLOS Glob Public Health. 2023 Mar 13;3(3):e0001664. doi: 10.1371/journal.pgph.0001664 (PMC10021758; doi:10.1371/journal.pgph.0001664)
Supplement: Attachment — Submitted filename: PGPH-D-22-01545 Response to Reviewers Comments.docx [file pgph.0001664.s004.docx]

**Journal Requirements Comments:**

1. Please amend your detailed Financial Disclosure statement. This is published with the article. It must therefore be completed in full sentences and contain the exact wording you wish to be published.

a) State the initials, alongside each funding source, of each author to receive each grant. For example: "This work was supported by the National Institutes of Health (####### to AM; ###### to CJ) and the National Science Foundation (###### to AM)."

b) State what role the funders took in the study. If the funders had no role in your study, please state: “The funders had no role in study design, data collection and analysis, decision to publish, or preparation of the manuscript.”

Response: This work was supported by funded by the National Institute for Health Research (NIHR) Global Health Research Unit on Improving Health in Slums (16/136/87 to RL, AO, EO, OF, JM) and Global Surgery (16/136/79 to RL) using UK aid from the UK Government to support global health research. The views expressed in this publication are those of the author(s) and not necessarily those of the NIHR or the UK Department of Health and Social Care. The funders had no role in study design, data collection and analysis, decision to publish, or preparation of the manuscript

2. Please update your online Competing Interests statement. If you have no competing interests to declare, please state: “The authors have declared that no competing interests exist.”

Response: The authors have declared that no competing interests exist

3. Please provide separate figure files in .tif or .eps format only and remove any figures embedded in your manuscript file. Please also ensure that all files are under our size limit of 10MB.

Response: Done

For more information about how to convert your figure files please see our guidelines: https://journals.plos.org/globalpublichealth/s/figures" https://journals.plos.org/globalpublichealth/s/figures

4. We have noticed that you have a list of Supporting Information legends in your manuscript. However, there are no corresponding files uploaded to the submission. Please upload them as separate files with the item type 'Supporting Information'.

Response: Done- we have attached files for supporting information

5. Please include copies of Supplementary Table 1 and Supplementary Table 2, which you refer to in your text on pages 7 and 14. Please upload them as separate files with the item type 'Supporting Information'. Please ensure that each Supporting Information file has a legend listed in the manuscript after the references list.

Response: Done. We uploaded them as supporting information files

6. We notice that your supplementary table (Appendix 1) is included in the manuscript file. Please remove them and upload them with the file type 'Supporting Information'. Please ensure that each Supporting Information file has a legend listed in the manuscript after the references list.

Response: Done.

**Reviewer #1:**

Abstract

a. Higher income and educational attainment were predictive of membership of this minority – It would be nice to have the inferential statistical values that affirms the prediction in brackets right after the sentence.

Response: The values are too complex to be captured in the abstract.

Background

b. Researchers estimate……... Is this “1.3-4 million cholera incidence” a global or national statistic?

Response: It is a global statistic and has been written into the sentence.

c. Kindly recast the Odds Ratios narrative in the introduction.

Response: The odds ratio narrative is part of a quote from a published study hence the necessary inclusion.

d. The gap in literature (and why DCE needs to be studied) has not been sufficiently captured.

Response: We have sufficiently captured this to the best of our knowledge. Thank you

Methods

e. The date (month and year) of data collection is not clearly stated anywhere – kindly provide dates of data collection.

Response: This has been added in the Survey sub-section.

f. This is one of the seven urban slums in the NIHR Global Health Research Unit on Improving Health in Slums Project [21] – Can authors provide the name of the slum please?

Response: For ethical reasons, the name of the slum was removed intentionally. A detailed description of the slum is provided in the referenced article.

g. A table that presents the details of the 48 IDI participants will be desirable.

h. It is not also clear where and how the authors acquired the telephone numbers of the 48 participants for the IDI. Were the numbers obtained from a directory or database?

Response: Phone numbers were obtained directly from participants who reside within the community and meet the eligibility criteria (gender and age). They are gatekeepers who are knowledgeable about health in their community.

i. It is preferred that the IDI excerpts be moved to the results section instead of them appearing in the materials and methods section.

Response: All previous studies have the IDI excerpts in the methods section as it was part of attribute and level identification procedures. We also considered it best practice to provide a summary of the qualitative aspect of the research design.

j. It would be desirable if the methods and results were reported in the past tense, considering that the research is completed and under review.

Response: This suggestion is well received. The method and result sections have been modified,

Reviewer #2: The manuscript has an interesting work and is well written. Observations from the methodological part are listed below.

1. Consent: Were the telephonic interviews were recorded? If so, was verbal consent also obtained for the recording? Please mention that as well.

Response: The interviews were recorded and verbal consent was obtained. This has been incorporated into the Attribute Selection sub section.

2. DCE research design: How were the IDI participants selected? whether the chosen participants had a history of either any of malaria, diarrhoea/cholera, or depression symptoms? Please eloquently discuss this.

Response: Participants were gatekeepers knowledgeable about health in their community and met the eligibility criteria. These are included in the Attribute Selection sub section.

3. Was there any inclusion or exclusion criteria for selecting participants for both IDI and for the DCE survey? A succinct description on this will be beneficial.

Response: This has been written into the *Participants and Sampling*, and pilot testing sub sections.

4. The first column in the fiig 1, is bit confusing can be re written as "Participants sampled from

respondents of household survey in Improving Health in Slums project".

Response: Thank you for the suggestion. Done.

Reviewer #3:

These are my charges to the team.

1. The sociodemographic are not well presented in a scientific fashion -I don’t like that table (opinion).

2. You tend to lump results that should be presented in a step wise or separate tables fashion for easy appreciation by readers. This can be done as follows.

a. clogit model.

b. -mlogit model for the presentation of the preferences of the respondents. Reporting of the beta coefficients.

c. -latent class analysis for the identification of the class phenotypes.

d. Predictors of the latent class membership

e. Willingness to pay analysis/ willingness to trade analysis

3. Reporting of the results text is missing the reporting of the beta-coefficients, their comparisons by level of strength and 95% confidence intervals. This is the main gist of a DCEs that you have completely at the mlogit and the wtp analysis.

Response: We accept that our presentation of results was overly aggregated and not ideally structured for readers to interpret the many scenarios and models we present. We thank the reviewer for their helpful suggestions on improving our presentation to provide a clearer, more step-wise journey through our results. Based on their recommendation, we have made the following changes

-	We have created sub-headings for our results section to provide the stepwise structure suggested by the reviewer.

-	We have disaggregated our tables by splitting model fit statistics into a separate table, separating results from the mixed logit and latent class models into separate tables, and placing class membership predictors for the latent class model into their table.

-	We have replaced odds ratios with β-coefficients for all results and expanded our results section with more detailed information on them.

-	We have chosen to keep the WTP results alongside the regression coefficients as we felt this made for easier exposition. However, we have incorporated all other suggestions above and do believe this has substantially improved our results section.

Additions:

The manuscripts cited in the manuscript are high quality and should guide the authors in generating a quality manuscript. Here is an article that I am currently reading and the other that has good content that the team can draw inspiration from. 1,2

Response: Thank you for the suggested articles.
